# On the Nature of Pulmonary Emphysema

**Published:** 1899-04-01

**Authors:** 


### ON THE NATURE OF PULMONARY EMPHYSEMA.

Dr. Samuel Gee has seldom been more convincing, both in matter and in form, than in the very careful Lumleian lecture which he has just delivered before the Royal College of Physicians on "The Nature of Pulmonary Emphysema." When considered entirely by itself this is a condition which may not seem to the average practitioner to be one of such importance as to be a matter of very great interest; indeed, to those practitioners who only see their patients when they are ill, and thus have but small concern in the management of their lives, emphysema no doubt assumes the character of a complication and aggravation of the illness about which they are consulted rather than as a condition which in itself demands, or, indeed, permits of treatment. And perhaps in out-patient practice and among working people, who hardly can alter the conditions of their lives, this may be a true conception of the position of affairs. But a careful consideration of Dr. Gee's arguments, and of the conclusions at which he arrives, is sufficient to show how largely it may be possible among the well-to-do, by arranging the mode of life, and by controlling the conditions which are the beginnings of emphysema, to prevent the occurrence of that disease. When one considers how serious an addition to the danger of many of the commonest chest diseases emphysema may be, and how largely its existence, even when uncomplicated, takes away the pleasures of life, one sees the importance of Dr. Gee's teaching that emphysema results from dyspnœa, and that, in the vast majority of cases, this is the result of chronic bronchitis, which, after all, is a more or less manageable disease. Dr. Gee begins by showing that a form of acute emphysema is produced in a very short time in cases of croup and of suffocative bronchitis, and taking croup more especially as his text, he shows that it is not the obstruction to the inspiration which is the direct cause of the emphysema, but rather the failure of the expiration to get rid of the air so drawn in. It is the lack of an expiratory power proportionate to the violent inspiratory efforts which are brought into play when there is any obstruction to the ingress of air, as, for example, in the case of croup, that leaves the lungs too full of air, and thus produces that over-distension which is the essence of emphysema. Inspiration is entirely due to muscular effort, and can be increased in response to the need for it, while expiration is largely due to elasticity, is, in fact, the elastic recoil to the efforts of inspiration, and if this elasticity is not sufficient, as when there is some obstruction to expiration, as in croup, or is overstrained as when inspiration is over deep, it falls short of what is required of it during an emergency of dyspnœa. Inspiration being the more important motor of the upper lung can draw air past the obstructing secretions, but of this air expiration can only expel a portion, and thus much air comes to be imprisoned in the parts which are, or which become emphysematous. In the second place, the elasticity of the lung is probably damaged. Normal inspiration may be compared to the drawing of a bow, and expiration to the bow's recoil, and it does not seem a very far-fetched similitude to compare the result of an enormous number of forced inspirations to a bow which has been over-drawn, bent, and crippled. The elastic recoil of the lungs is weakened and the air cells become gradually and even rapidly distended. Now the same sequence seems to occur in cases of chronic emphysema. That is to say, the condition is induced by the action of a slight but an oft-repeated difficulty of breathing, set up in the majority of cases by bronchitis. A man about middle life begins to suffer from slight shortness of breath on exertion. His breathlessness increases slowly and insidiously until his breath is always short, but no signs of dyspnœa, no laborious or extraordinary movements of respiration are apparent so long as he is at rest, nor until he makes some effort, such as that of talking or of moving the body. In cases still worse the dyspnœa persists even when the patient is at rest. The dyspnœa of emphysema, like most other kinds of permanent dyspnœa, is liable to paroxysms, in which the shortness of breath becomes very distressing. These paroxysms are produced by any effort, and will even sometimes occur during sleep—will suddenly awaken the patient or will occur just as he is falling off to sleep. The mere taking of food may be enough to provoke an attack, and so may any sudden change of the temperature of the air. Now suppose a patient suffering in this way, and in whom the physical signs of emphysema are apparent, has no cough and no expectoration, we might well ask whether he were not the subject of protopathic emphysema; and some physicians say that they have met with such patients, and that, therefore, they believe in the occurrence of emphysema, pure, simple, and uncomplicated. Dr. Gee, however, says that this has not been his experience. He has never known this progressive dyspnœa to be unattended by cough and expectoration. The cough is a tickling cough brought on by a troublesome sensation referred to the larynx, while the expectoration is very scanty and consists of pure mucus. Now if we admit that a man in a state of perfect health ought not to cough or expectorate, and also that expectoration implies catarrh, it follows that at no period in the progress of emphysema is that condition unassociated with catarrh. We may, then, conclude that pulmonary emphysema is chiefly due to forced inspiration, although forced expiration may to some small extent play its part; that the forced inspiration is rendered necessary by a feeling of dyspnœa; that the dyspnœa which occurs at the beginning of pulmonary emphysema, and which determines it, is consequent upon obstruction of the air passages; that in chronic progressive emphysema this obstruction depends upon bronchitis, either humid and attended with free secretion, or dry with scanty secretion; that when once emphysema is set up the necessity for forced inspiration is increased by the natural defect of expiratory power in the lungs and in certain portions of the lungs; and that the degeneration and atrophy of lung tissue met with in emphysema are usually dependent upon preceding over-distension. The importance of these generalisations are obvious. Well-established emphysema is a condition of atrophy and degeneration for which, perhaps, we can do nothing; but when we recognise its dependence upon a precedent bronchitis or frequently recurring catarrh this precedent condition attains a position of importance in our eyes which it might not otherwise possess.

